# Extracts of *Phellinus linteus*, Bamboo (*Sasa senanensis*) Leaf and Chaga Mushroom (*Inonotus obliquus*) Exhibit Antitumor Activity through Activating Innate Immunity

**DOI:** 10.3390/nu12082279

**Published:** 2020-07-29

**Authors:** Jun Fang, Shanghui Gao, Rayhanul Islam, Yuji Teramoto, Hiroshi Maeda

**Affiliations:** 1 Faculty of Pharmaceutical Sciences, Sojo University, Kumamoto 860-0082, Japan; gaoshanghui94@gmail.com (S.G.); rayhanulislam88@gmail.com (R.I.); 2 Department of Applied Microbial Technology, Faculty of Biotechnology and Life Science, Sojo University, Kumamoto 860-0082, Japan; yuji@bio.sojo-u.ac.jp; 3 Department of Microbiology, Graduate School of Medical Sciences, Kumamoto University, Kumamoto 860-8556, Japan; 4 Biodynamics Research Foundation, Kumamoto 862-0954, Japan

**Keywords:** *Phellinus linteus*, Kumaizasa, bamboo leaf, Chaga mushroom, cancer prevention, macrophage, immunostimulation, innate immunity, supplement

## Abstract

Natural products have attracted great interest for some time as alternative methods against cancers by fulfilling immunomodulating properties. In this study, we investigated the activity of hot water extracts (120 °C, >30 min) of *Phellinus linteus,* fresh leaves of Kumaizasa bamboo and Chaga mushroom which we called MeshimaMax, for cancer prevention and treatment by using different solid tumor models. In the implanted mouse sarcoma S180 tumor, MeshimaMax treatment significantly inhibited tumor growth when it was applied at the early stage of tumor inoculation. The effect was further confirmed by using carcinogen induced tumors, i.e., azoxymethane (AOM)/dextran sulfate sodium (DSS) induced mouse colon cancer and 7,12-dimethylbenz anthracene (DMBA) induced rat breast cancer. In both cases the occurrences of tumors were remarkably suppressed by administration of MeshimaMax which consists of three components above. More importantly, when MeshimaMax was combined with an anticancer chemotherapeutic drug, the therapeutic effect was remarkably improved. In vitro studies showed that when MeshimaMax was applied to mouse macrophage RAW264.7 cells the phagocytosis of macrophages was significantly activated, which was evaluated by using living yeast cells as well as synthetic nanoparticles. A cytotoxicity assay showed the 50% inhibitory concentration (IC_50_) was higher than 1 mg/mL and normal cells were 2–3 times more tolerant to MeshimaMax than cancer cells. These findings suggest the potential application of MeshimaMax for cancer prevention and as supplement regimen for anticancer chemotherapy, probably functioning through activation of innate immunity, which may benefit cancer patients as an alternative supplement.

## 1. Introduction

Cancer remains the major cause of human death in the world. Many factors and molecules are involved in the initiation, promotion and progression of cancer. One of the causal factors of tumor progression is the suppression of immune functions, namely the innate and acquired immunity. When fighting against tumor cells, this suppression means that immune functions cannot operate properly and their activities are reduced [1]. Restoring or activating immune competence can thus be one of the primary strategies for active anticancer therapy. Immunotherapy, as an important anticancer modality, has received considerable attention and many immunotherapeutic drugs have been developed and used in clinics. Additionally, the use of alternative agents from natural products as immunomodulators against cancers, have also attracted great interest in the past [2,3].

Polysaccharides from mushrooms and microbial cell-wall components have been shown to be efficient immunomodulators. It has also been reported that extracts of Kumaizasa bamboo (*Sasa senanensis*) leaves demonstrated anti-inflammatory properties [4] and our previous studies clearly showed the strong effect of these bamboo leaf extracts to activate macrophages and neutral killer (NK) cells, inducing the production of proinflammatory cytokines, e.g., interleukin-12 (IL-12) and nitric oxide (NO), consequently preventing carcinogenesis and inhibiting tumor growth [5].

*Phellinus linteus* is a medicinal mushroom that has been used in traditional medicine in China, Japan and Korea for centuries as an ailment drug for many diseases, such as gastroenteric dysfunction, diarrhea, hemorrhage and cancers. The activity of *Phellinus linteus* against cancer has been reported in many studies using different cancers and it is thus considered as a promising anti-cancer agent [6,7,8]. Though the mechanisms behind its anti-cancer activity are not fully clear, a previous study using extracts from fruit-bodies and the mycelium of *Phellinus linteus* suggested that it could stimulate the hormonal and cell-mediated immune function, in addition to modulating the inflammatory reactions caused by a variety of stimuli, consequently suppressing tumor growth and metastasis [9].

The Chaga mushroom (*Inonotus obliquus*) is a type of fungus that grows mainly on the bark of birch trees in cold climates, such as Northern Europe, Siberia, Russia, Korea, Northern Canada and Alaska. It has been used in traditional medicine for centuries to treat various health problems, especially by Khanty people. In China, Korea, Japan, Russia, and the Baltics, extracts of Chaga mushroom have been used because of their favorable effects, for example, promoting lipid metabolism and modulating cardiac function, as well as antibacterial, anti-inflammatory, antioxidant, and antitumor activities [10,11,12]. To date, several compounds have been identified from the Chaga mushroom, such as polysaccharides, triterpenes, polyphenols, betulin and betulinic acid, which are considered to be responsible for most of its therapeutic activities [10,11,12].

Hot water extraction method is commonly used to obtain extracts from plants, however the efficacy and yield are relatively low. Previously we modified the extraction method using vigorous steam conditions at high temperature and high pressure (vigorous extract) for Kumaizasa (*Sasa senanesis*) leaves, of which the extracts contained a larger quantity of active compounds than conventional hot-water extracts [5]. Along this line, an extract mixture of *Phellinus linteus,* Kumaizasa bamboo and Chaga mushroom using vigorous steam conditions was prepared. In this study, we investigated the activity of this extract mixture (MeshimaMax) for cancer prevention and treatment by using different solid tumor models, including chemical carcinogen induced cancer. The possible mechanisms involved in the activity of the extracts was examined especially focusing on their potential to stimulate innate immunity.

## 2. Materials and Methods 

### 2.1. Materials

Dextran Sulfate Sodium Salt (DSS) was purchased from MP Biomedicals, LLC (Irvine, CA, USA). Latex beads-Rabbit IgG-FITC Complex was from Cayman chemical (Ann Arbor, MI, USA). Thiazolyl Blue Tetrazolium Bromide (MTT) was purchased from Sigma-Aldrich Chemical (St. Louis, MO, USA). Azoxymethane (AOM), 7,12-dimethylbenz anthracene (DMBA), cell culture medium (RPMI-1640, DMEM, DMEM:F12) were purchased from Fujifilm Wako Pure Chemical Corporation (Osaka, Japan). MeshimaMax was provided by ACSYS UN (Tokyo, Japan). Anticancer nanomedicine HPMA copolymer conjugated pirarubicin (P-THP) was synthesized in our laboratory as reported previously [13].

### 2.2. Animals

ddY mice (male, 6-weeks old), ICR mice (male, 5-weeks old) and SD rats (female, 6-weeks old) were bought from SLC (Shizuoka, Japan). They were housed in groups of four or five per cage and were maintained at 22 ±10 °C and 55% ±5% relative humidity. Lighting was automatic on a 12-h light/dark cycle. All experiments were carried out according to the Laboratory Protocol of Animal Handling, Sojo University. Ethical approval was given by Sojo University, Animal Ethical Committee (decision no. P/009/2019) in 2019.

### 2.3. Cell Lines

RAW264.7 cells of mice macrophages were purchased from ATCC (Manassas, VA). Human ovarian cancer A2780 cells were distributed by Professor Kobayashi, Faculty of Medicine, Kagoshima University. Human cervical cancer HeLa cells were from Riken cell bank (Tsukuba, Japan). Mouse hepatocytes AML12 were kindly provided by Dr. Zhang of Department of toxicology, Anhui Medical University, China. Monkey kidney epithelial cells (CCL-81) and yeast (*Saccharomyces cerevisiae*) cells were kindly provided by Professor Yokomizo, Microbiology Laboratory, Faculty of Pharmaceutical Sciences, Sojo University. RAW264.7 cells were cultured in DMEM (high glucose) containing 10% fetal bovine serum (FBS), CCL-81 cells and A2780 cells were maintained in RPMI-1640 containing 10% FBS. HeLa cells were cultured in DMEM containing 10% FBS and AML12 cells were cultured in DMEM/F12 medium containing 10% FBS with 10 µg/mL insulin, 5.5 µg/mL transferrin, 5 ng/mL selenium, and 40 ng/mL dexamethasone. All of the cells were cultured at 37 °C with 5% CO_2_.

### 2.4. Antitumor Effect of MeshimaMax

The potential effect of MeshimaMax on tumor growth and carcinogenesis was investigated in various murine solid tumor models, including an AOM/DSS induced mouse colon cancer model, a DMBA induced rat breast cancer model and a mouse sarcoma S180 subcutaneously transplanted model.

AOM/DSS induced mouse colon cancer model was prepared by administering AOM (10 mg/kg) intraperitoneally into ICR mice, after 1 week, 2% DSS was added into drinking water and applied to the mice for 1 week. In the MeshimaMax treatment group, different concentrations of MeshimaMax (0.03%, 0.1%, 0.3%) were applied to the mice as drinking during the entire experiment, from the time of AOM administration, during which it was mixed in the food when DSS was applied through drinking water. Twelve weeks after DSS administration, the mice were sacrificed and the number of colon cancers and the diameter of tumor nodules were measured.

The DMBA induced rat breast cancer model was established by oral administration of DMBA (10 mg/mL/rat) which was dissolved in corn oil. For the treatment, MeshimaMax (0.03%, 0.1%, 0.3%) was added to the drinking water of rats during the entire experiment. During the experiment, the tumor development status was observed regularly and the tumor size and number were measured.

The mouse S180 tumor model was prepared by injecting the cancer cells (2 × 10^6^/100 uL) subcutaneously into the dorsal skin of a ddY mouse. MeshimaMax (0.03%, 0.1%, 0.3%) was added to the drinking water of mice for treatment. In some experiments, MeshimaMax was administered from the day that the S180 cells were transplanted (preventive administration). In another set of experiments, MeshimaMax was applied one week after the transplantation of S180 cells when the diameter of the tumor had reached 6–8 mm (therapeutic administration). In a separate experiment, MeshimaMax was combined with anticancer nanomedicine P-THP, in which P-THP was injected i.v. 7 days after the inoculation of S180 cells, 0.1% MeshimaMax was then administered as drinking water till the end of the experiment. During the experiment, the tumor regression or growth was observed periodically and tumor size was measured.

### 2.5. Cytotoxicity Assay

The cytotoxicity of MeshimaMax was investigated in cancer cells (A2780, HeLa) as well as normal cells (CCL-81, AML12) by MTT assay. Briefly, Cells were seeded in 96-well plates (3000 cells/well), after overnight preincubation, MeshimaMax of difference concentrations was added and the cells were further cultured for 48 hours. Then, the medium was removed, and MTT was added to the final concentration of 0.56 mg/mL. After incubation for 4 hours at 37 ^°^C, 5% CO_2_, the medium was removed, followed by adding 150 ul DMSO to each well. The cell viability was calculated by measuring the optical density (OD) at 570 nm.

### 2.6. Effect of MeshimaMax on Phagocytosis of Macrophages

The effect of MeshimaMax on phagocytosis of macrophages was examined by using RAW264.7 cells. Cells (1 × 10^5^/1.5 mL) were seeded on a 6-well plate, after overnight preincubation, MeshimaMax was added at different concentrations and the cells were cultured for 24 hours. Then, yeast cells (3 × 10^5^ cells) were administered to the macrophage culture system. The macrophages incorporating yeast were observed and counted with a microscope (BZ-X700, Keyence Co. Ltd., Osaka, Japan). Two-hundred RAW264.7 cells were counted and the phagocytic ability of macrophages was calculated by the following equation, phagocytosis = A / 200 × 100%, in which A was the number of macrophages which took up yeast cells.

In a separate experiment, fluorescence nanoparticles (Latex beads-Rabbit IgG-FITC complex) were used instead of yeast, in which the phagocytosed nanoparticles were quantified by flow cytometry (Accuri^™^ C6 Plus, BD, Franklin Lakes, New Jersey, USA).

### 2.7. Statistical Analyses

All data were expressed as means ±SD. Data were analyzed using ANOVA followed by the Bonferroni multiple comparison test. A difference was considered statistically significant when *P* < 0.05.

## 3. Results

### 3.1. Effect of MeshimaMax on Carcinogenesis and Tumor Growth

#### 3.1.1. MeshimaMax Inhibits Colon Cancer Carcinogenesis Induced by AOM/DSS: Cancer Preventive Effect

**Figure 1:**
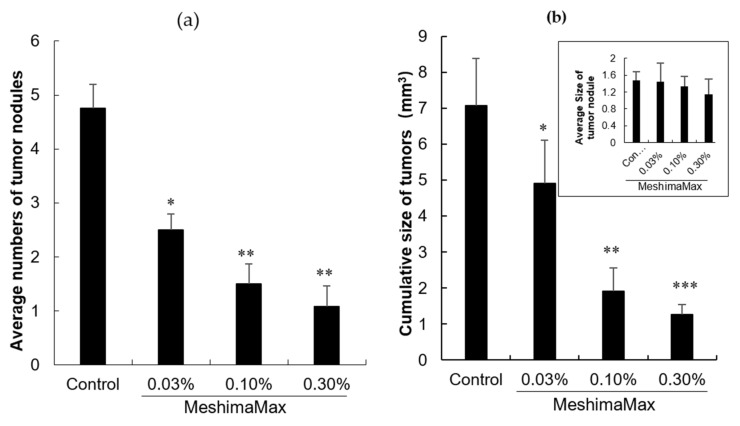
Inhibitory effect of MeshimaMax on the tumorigenesis in mice treated by azoxymethane (AOM)/dextran sulfate sodium (DSS). (**a**) Average numbers of tumor nodules in the colon; (**b**) Cumulative size of tumor nodules in each mouse. Inset of (b) shows the average size of tumor nodules developed in the colon of mice. * *P* < 0.05; ** *P* < 0.01; *** *P* < 0.001 vs Control. Data are mean ± SD, *n* = 6–8.

The effect of MeshimaMax against cancer was first examined in an AOM/DSS induced mouse colon cancer model, where multiple colon cancer nodules appeared in all of the mice (Figure 1a). MeshimaMax treatment significantly decreased the numbers of tumor nodules in a dose-dependent manner (Figure 1a). However, as for the sizes of the formed tumor nodules, no apparent changes were observed between the MeshimaMax treatment group and the control group (Figure in set of 1b). However, the cumulative size of tumors in each mouse treated by MeshimaMax was remarkably decreased (Figure 1b).

#### 3.1.2. Suppression of DMBA Induced Breast Cancer Carcinogenesis in Rat by MeshimaMax: Cancer Preventive Effect

Next, we investigated the potential antitumor activity of MeshimaMax in another carcinogenesis model, i.e., DMBA induced rat breast cancer model, in which tumors developed from 10 weeks after AOM administration and tumors appeared in all the mice after 20 weeks (Table 1). MeshimaMax treatment remarkably delayed the occurrence of tumors, no tumors developed up to 15 weeks after DMBA administration (Table 1). At the time of 100% tumor occurrence in the control group, MeshimaMax treated rats showed a 60% occurrence of tumor (Table 1). The tumor incidences after 100 days were 83% for control group and 0% for MeshimaMax treatment at either 0.03% or 0.1% (Table 1).

**Figure 2:**
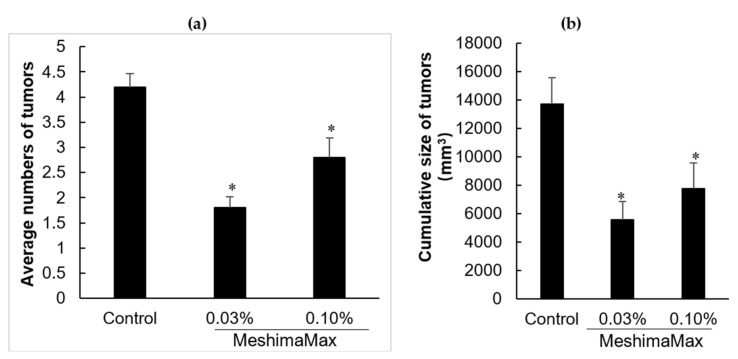
Suppression of tumor development by MeshimaMax in DBMA treated rats. (**a**) Average numbers of breast tumors developed; (**b**) Cumulative size (tumor volume) of breast tumors. Data are those at day 206 after MeshimaMax treatment. * *P* < 0.05 vs Control. Data are mean ± SD, *n* = 5–6.

Figure 2 shows the numbers of breast tumors developed in the rats and the cumulative tumor sizes at 206 days after DMBA administration. Similar to the results in AOM/DSS induced colon cancer model (Figure 1), rats taking MeshimaMax exhibited significantly lower numbers of tumors than control rats (Figure 2a), the cumulative tumor size in each rat treated by MeshimaMax was also significantly decreased, though no linear dose-dependency was observed (Figure 2b).

#### 3.1.3. Antitumor Effect MeshimaMax in S180 Transplanted Tumor Model

**Figure 3:**
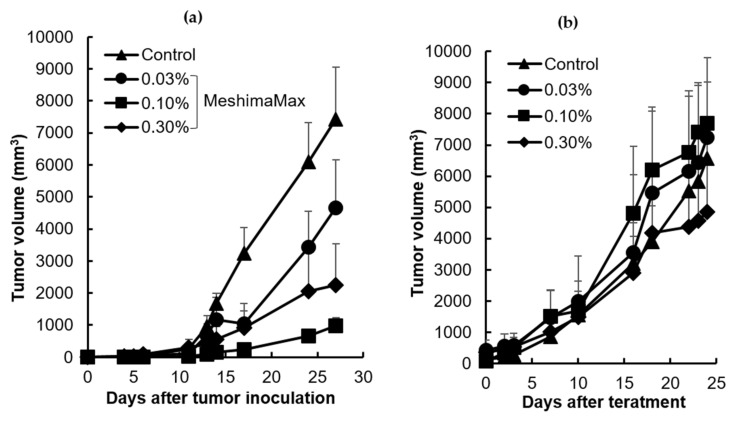
Antitumor effect of MeshimaMax against mouse sarcoma S180 solid tumor. (**a**) MeshimaMax treatment was carried out from the day of S180 tumor cell inoculation; (**b**) MeshimaMax treatment was carried out from day 7 after S180 tumor cell inoculation when tumor had grown to 7–8 mm in diameter. Data are mean ± SD, *n* = 4–8.

Finally, we examined the antitumor effect of MeshimaMax in a mouse sarcoma S180 solid tumor model, where the S180 cells were injected into the dorsal skin of mice to form tumors. MeshimaMax treatment was carried out at different time points. When it was applied from the same day of tumor cell inoculation, significant delay of tumor growth was observed (Figure 3a). However, when MeshimaMax treatment started 7 days after tumor inoculation, when the tumor had reached 7–8 mm in diameter, no apparent suppression of tumor growth was observed (Figure 3b).

#### 3.1.4. Improved Anticancer Effect by MeshimaMax in Combination with Anticancer Nanomedicine

**Figure 4:**
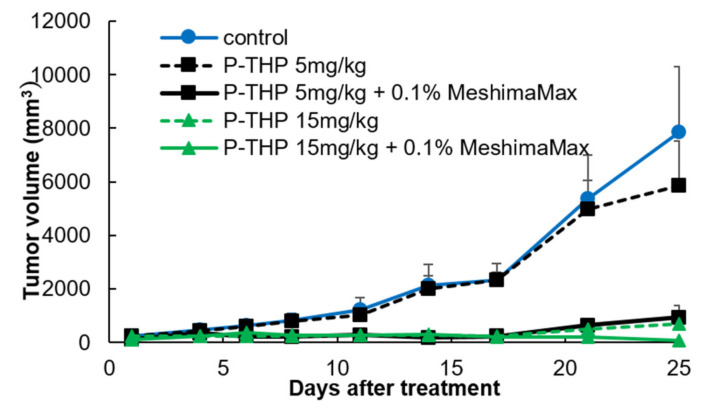
Antitumor effect of MeshimaMax in combination with anticancer nanomedicine HPMA copolymer conjugated pirarubicin (P-THP), against mouse sarcoma S180 solid tumor. MeshimaMax treatment was carried out from day 7 after S180 tumor cell inoculation when the tumor had grown to 7–8 mm in diameter and P-THP was injected i.v. at day 7 after tumor inoculation. Data are mean ± SD, *n* = 4–5.

To further investigate the application potential of MeshimaMax for cancer, we carried out a combination therapy using MeshimaMax with anticancer nanomedicine P-THP. As shown in Figure 4, P-THP exhibited a dose-dependent antitumor effect, where no apparent tumor suppression was observed at the dose of 5 mg/kg. Importantly, though MeshimaMax alone did not show a significant therapeutic effect (Figure 3b), however, when it was combined with P-THP (5 mg/kg), a remarkable antitumor effect was achieved comparable to that of P-THP at 15 mg/kg where no apparent growth of tumor was found up to 25 days after treatment (Figure 4). Moreover, when 15 mg/kg P-THP was combined with MeshimaMax, a significant decrease in tumor size was observed (Figure 4), in which three out of five mice were free of tumors 25 days after treatment suggesting a complete cure of tumors.

### 3.2. Cytotoxicity of MeshimaMax

**Figure 5:**
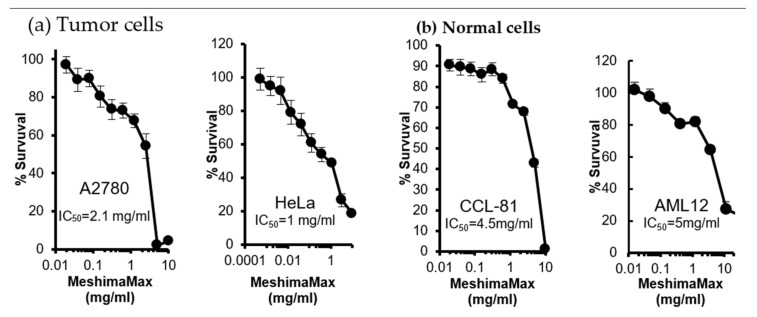
In vitro cytotoxicity of MeshimaMax to different cells. (**a**) Tumor cells: human ovarian cancer A2780 cells and human cervical cancer HeLa cells. (**b**) Normal cells: monkey kidney epithelial cells CCL-81 and mouse hepatocytes AML12.

The results described above suggest the inhibitory effect of MeshimaMax on tumor development and progression. We then investigated the possible mechanisms involved in the antitumor effect of MeshimaMax. First, we examined the in vitro cytotoxicity of MeshimaMax against tumor cells, as well as normal cells. As shown in Figure 5, the 50% inhibitory concentrations (IC_50_) of MeshimaMax against tumor cells (i.e., A2780 and HeLa) were 2–5 times lower than those against normal cells (i.e., CCL-81 and AML12), indicating a higher cytotoxicity of MeshimaMax to tumor cells than to normal cells.

### 3.3. Effect of MeshimaMax on Phagocytosis of Macrophages

**Figure 6:**
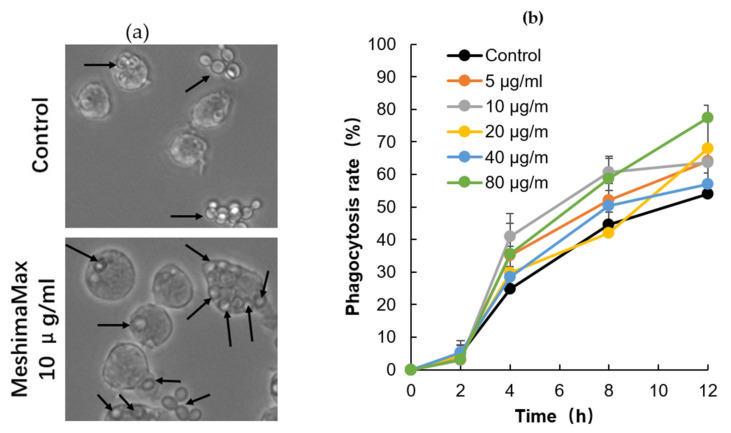
Increase in macrophage phagocytotic activity by MeshimaMax. (**a**) Engulfment of yeast by macrophages in the presence (10 µg/mL)/absence (control) of MeshimaMax at 12 h after adding yeast to macrophages, arrows indicate the yeast engulfed by macrophages. In control macrophages without MeshimaMax treatment, less yeast was internalized into macrophages and more yeast stayed outside macrophages, whereas after MeshimaMax treatment, much more yeast was taken up by macrophages. (**b**) Phagocytosis rate of macrophages as calculated from the results shown in (**a**). See text for details.

To investigate the mechanisms of MeshimaMax induced tumor suppression, we focused on its effect on the activation of macrophages and examined the phagocytotic activity using RAW264.7 cells. As shown in Figure 6, when yeast was applied to macrophages, it was taken up by macrophages gradually in a time-dependent manner, after 12 h more than 50% of macrophages exhibited phagocytotic activity (uptake of yeast). More importantly, MeshimaMax treatment significantly enhanced the phagocytosis of macrophages, though no dose-dependency was observed (Figure 6b). At 12 h after the addition of yeasts in the presence of MeshimaMax at 80 µg/mL, the macrophages showing phagocytotic activity increased to more than 75% which was 25% higher than macrophages without MeshimaMax treatment (Figure 6b).

**Figure 7:**
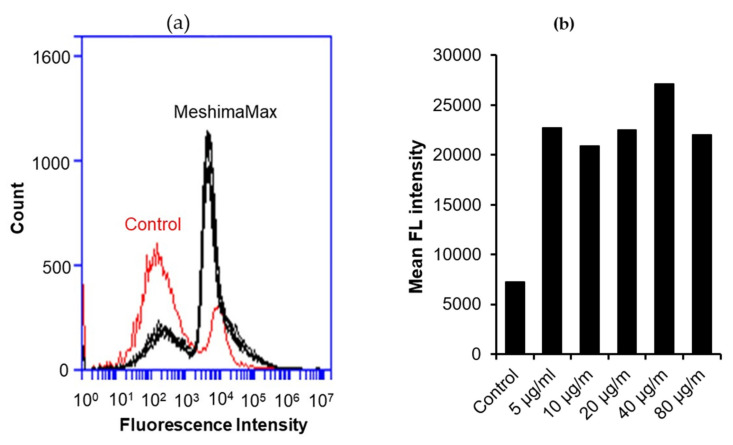
Internalization of fluorescence nano-beads by macrophages under the treatment of MeshimaMax. The flow cytometry results are shown in (**a**) where MeshimaMax treatment induced a remarkable shift to a higher fluorescence intensity than untreated controls indicating that more nano-beads were taken up by macrophages. The mean fluorescence intensities of macrophages treated by different concentrations of MeshimaMax are shown in (**b**). See text for details.

Further, we confirmed this effect by using fluorescence nanoparticles, where the number of nanoparticles incorporated into macrophages is presented by the fluorescence intensity using flow cytometry. As a result, as compared with the control group without MeshimaMax treatment, the number of particles taken in by macrophages treated by MeshimaMax increased significantly (Figure 7a). The average fluorescence intensity in the cells treated with MeshimaMax, which indicated the amount of the phagocytic particles, increased to more than 3 times that of the control cells at a dose of 0.04 mg/mL. Higher doses of MeshimaMax (i.e., 0.08 mg/mL) showed a slight decrease in fluorescence intensity, indicating an inverted “U-shaped” dose-effect (Figure 7b).

## 4. Discussion

In a previous study, we reported the antitumor activity of bamboo (*Sasa senanensis*) leaf extracts through immunopotentiation, i.e., inducing the production of inflammatory cytokines and activating macrophages and NK cells [5]. Such natural products from plants with immunostimulating activity provide an alternative or supplemental anticancer regimen. They may particularly benefit those who are in an immunosuppressed state, such as cancer patients during and after chemotherapy and older populations. Investigation and development of such supplemental nutrients is thus of great importance. Along this line, in this study, we first demonstrated that MeshimaMax, the extract mixture of *Phellinus linteus*, bamboo (*Sasa senanensis*) leaf and Chaga mushroom *(Inonotus obliquus*), exhibited a cancer preventive effect, which was probably at least partly through the activation of macrophages—the innate immunity.

The antitumor effect of MeshimaMax was considered best for tumor prevention, but not direct tumor killing. When a tumor grew to a palpable size, administration of MeshimaMax did not show a significant effect on tumor growth suppression (Figure 3b). This was not consistent with our previous study using bamboo leaf extracts [5], which may partly due to the different sizes of tumors at the starting point, the tumors were 6–8 mm in the present study which was larger than those used in the previous study (5–6 mm in diameter). However, more importantly when MeshimaMax was applied to the mice at the same time of tumor inoculation, remarkable suppression of tumor growth was achieved (Figure 3a). To confirm the cancer preventive effect of MeshimaMax, we carried out experiments using carcinogen induced tumor models, i.e., AOM/DSS induced mouse colon cancer model and DMBA induced rat breast cancer model, which are optimal models to mimic the initiation and progression of human cancer. In both models, we found the formation and growth of tumors were significantly inhibited by MeshimaMax (Figure 1 and Figure 2). It should also be noted that breast cancer is the second most common cancer, after lung cancer, in the world and the leading cause of cancer deaths in women [14], while colon cancer has recently become the second most common cause of cancer in women and the third most common in men [15]. Our current results may thus offer options to prevent the incidence of those cancers, especially for those of high risk. The cancer preventive effect of MeshimaMax was similarly observed in chemical carcinogenesis in both breast cancer and colon cancer, as well as transplanted mouse sarcoma, suggesting the effect is not tumor type-specific, but is applicable to many types of cancers which warrants further investigations. More importantly, though MeshimaMax alone did not show a direct antitumor effect, the combination therapy of MeshimaMax with anticancer drug P-THP significantly improved the therapeutic effect of P-THP (Figure 4), suggesting its potential application as a supplement regimen during anticancer chemotherapy.

Regarding the mechanisms of MeshimaMax on cancer prevention, along with our previous study using bamboo (*Sasa senanensis*) leaf extracts [5], we focused on its potential for activation of innate immunity and investigated its effect on macrophage activation by examining the phagocytotic activity of macrophages. As expected, we found significantly increased phagocytosis of cultured macrophages under the treatment of MeshimaMax, which was demonstrated by using both living yeast and the FITC-labeled latex nanoparticles (Figure 6 and Figure 7). Phagocytosis is one of the most important functions of macrophages against microbial, foreign bodies and damaged cells including cancer cells in innate immunity. It is thus reasonable that MeshimaMax fulfills its cancer preventive effect through macrophage activation. It has been also known that macrophages have two major activation phenotypes upon different stimuli. Firstly, macrophage polarization, where M1 macrophages show mostly inflammatory properties excreting inflammatory cytokines such as IL-2, IL-6, inducible NO synthase and having high phagocytotic activity to clear microbial and damaged cells. Secondly, M2 macrophages exhibit mainly anti-inflammatory effects by generating anti-inflammatory cytokines such as IL-10, a transforming growth factor for supporting tissue repair [16]. We thus consider that MeshimaMax may induce the activation of macrophages towards M1 polarization, which will be a forthcoming challenge in our future study. Many studies have reported the versatile effects of *Phellinus linteus* and its bioactive components including anti-inflammatory activity, anti-oxidative activity, anti-microbial activity and anticancer activity etc., in which immunomodulation is one of the critically important mechanisms involved in its functions [17]. *Phellinus linteus* could enhance the expression and activation of macrophages and NK cells, upregulating the innate immunity [18,19], our present study confirmed this notion. *Phellinus linteus* is also known to increase the proliferation and activity of antigen presentation cells, B lymphocytes, as well as T lymphocytes [20,21,22], suggesting its effect on acquired immunity. Accordingly, the immunoregulating effect of MeshimaMax may also include multiple mechanisms, the forthcoming comprehensive study will focus in depth on the immunomodulating mechanisms of MeshimaMax.

However, in this study, we found the effect of MeshimaMax was not in a linear dose-dependent manner in either in vivo (Figure 1, Figure 2 and Figure 3) or in vitro studies (Figure 6 and Figure 7). Relatively lower concentrations (e.g., 0.03%, 0.1%) showed better effects whereas higher concentrations (e.g., 0.3%) resulted in no significant increase in effect (Figure 1) or, on the contrary, a decreased effect (Figure 2 and Figure 3). Actually, similar results were reported in our previous studies using Kumaizasa bamboo leaf extract [5]. Though the present study and our previous study [5] strongly suggest the immunostimulating, proinflammatory activity of the tested extracts, an earlier study also reported the anti-inflammatory effect of bamboo leaf extract [4]. Accordingly, we considered that the effect of MeshimaMax, similar to Kumaizasa bamboo leaf extract, may be an inverted “U-shaped” dose-effect, which low or moderate concentrations exhibit proinflammatory activity or M1 polarization of macrophages, whereas higher doses may inhibit inflammatory reaction pushing the macrophages to M2 polarization. Such “U-shaped” or convex “U-shaped” dose-effects are common biological events, particularly for chemicals with hormonal activities [23]. However, the detailed mechanisms involved in the dose-effect in the present study are not clear and further investigations are warranted to elucidate this issue. More importantly, we also found the dose responses varied in different models or animal species, for example, SD rat models seemed to respond at much lower doses (i.e., 0.03%) (Figure 1), whereas balb/c mice showed almost a linear effect up to 0.3% of MeshimaMax (Figure 2), these findings may reflect the different immune responses of different species of animals, which should be considered in future studies. In other words, overdosing of immunopotentiator may exhibit opposite functions.

Additionally, in this study, we also examined the cytotoxicity of MeshimaMax on cancer cells, as well as normal cells. Here, the IC_50_ was more than 1 mg/mL which is an extremely high concentration compared with that of macrophage activation (Figure 5). This suggests that MeshimaMax does not have an apparent direct antitumor effect. However, interestingly, the IC_50_ against normal cells was 2–3 times higher than that of cancer cells (Figure 5). This finding indicates that normal cells are more tolerant to MeshimaMax, namely MeshimaMax is a friendly, non-toxic supplement to our bodies.

## 5. Conclusions

In this study, we reported that MeshimaMax, the extract mixture of *Phellinus linteus*, bamboo (*Sasa senanensis*) leaves and Chaga mushroom *(Inonotus obliquus*), could potentially inhibit the initiation of carcinogenesis and the growth of tumors. Thus, MeshimaMax could potentially show a cancer preventive effect, which was supported by studies not only in transplanted tumor models, but also in carcinogen induced tumor models. This effect was attributed to the activation of macrophages, which is the most important component of innate immunity. MeshimaMax also showed almost no cytotoxicity, especially to normal cells. MeshimaMax may thus become an alternative supplemental nutrient for patients suffering from cancer and a maintenance modality for patients, post chemotherapy.

## Figures and Tables

**Table 1:** Incidence of breast tumor in rats receiving 7,12-dimethylbenz anthracene (DMBA) in the presence/absence of MeshimaMax.

Days after DMBA Administration	Incidence of Breast Tumor (No. of Rats with Tumor/Total No. of Rats)		
	Control	MeshimaMax 0.03%	MeshimaMax 0.1%
73	1/6	0/5	0/5
87	2/6	0/5	0/5
93	5/6	0/5	0/5
107	4/5*	0/5	0/5
108	4/5 *	1/5	2/5
115	4/5 *	1/5	3/5
122	4/5 *	1/5	3/5
132	4/5 *	3/5	3/5
140	5/5 *	3/5	3/5

* One rat died of cancer.

## References

[1] Schreiber R.D., Old L.J., Smyth M.J. (2011). Cancer immunoediting: Integrating immunity’s roles in cancer suppression and promotion. Science.

[2] Dutta S., Mahalanobish S., Saha S., Ghosh S., Sil P.C. (2019). Natural products: An upcoming therapeutic approach to cancer. Food Chem. Toxicol..

[3] Li Y., Li S., Meng X., Gan R.Y., Zhang J.J., Li H.B. (2017). Dietary Natural Products for Prevention and Treatment of Breast Cancer. Nutrients.

[4] Shibata M., Yamatake Y., Sakamoto M., Kanamori M., Takagi K. (1975). Pharmacological studies on bamboo grass (1): Acute toxicity and anti-inflammatory and antiulcerogenic activities of water-soluble fraction (Folin) extracted from Sasa albomarginata Makino et Shibata. Nippon Yakurigaku Zasshi (Jpn.).

[5] Seki T., Kida K., Maeda H. (2010). Immunostimulation-Mediated Anti-tumor Activity of Bamboo (*Sasa senanensis*) Leaf Extracts Obtained Under ‘Vigorous’ Condition. Evid. Based Complement Altern. Med..

[6] Lu T.L., Huang G.J., Lu T.J., Wu J.B., Wu C.H., Yang T.C., Iizuka A., Chen Y.F. (2009). Hispolon from *Phellinus linteus* has antiproliferative effects via MDM2-recruited ERK1/2 activity in breast and bladder cancer cells. Food Chem. Toxicol..

[7] Sliva D., Jedinak A., Kawasaki J., Harvey K., Slivova V. (2008). *Phellinus linteus* suppresses growth, angiogenesis and invasive behaviour of breast cancer cells through the inhibition of AKT signaling. Br. J. Cancer.

[8] Zhu T., Kim S.H., Chen C.Y. (2008). A medicinal mushroom: *Phellinus linteus*. Curr. Med. Chem..

[9] Lee Y.S., Kang Y.H., Jung J.Y., Lee S., Ohuchi K., Shin K.H., Kang I.J., Park J.H., Shin H.K., Lim S.S. (2008). Protein glycation inhibitors from the fruiting body of *Phellinus linteus*. Biol. Pharm. Bull..

[10] Balandaykin M.E., Zmitrovich I.V. (2015). Review on Chaga Medicinal Mushroom, *Inonotus obliquus* (Higher Basidiomycetes): Realm of Medicinal Applications and Approaches on Estimating its Resource Potential. Int. J. Med. Mushrooms.

[11] Duru K.C., Kovaleva E.G., Danilova I.G., van der Bijl P. (2019). The pharmacological potential and possible molecular mechanisms of action of *Inonotus obliquus* from preclinical studies. Phytother. Res..

[12] Song F.Q., Liu Y., Kong X.S., Chang W., Song G. (2013). Progress on understanding the anticancer mechanisms of medicinal mushroom: *Inonotus obliquus*. Asian Pac. J. Cancer Prev..

[13] Nakamura H., Etrych T., Chytil P., Ohkubo M., Fang J., Ulbrich K., Maeda H. (2014). Two step mechanisms of tumor selective delivery of N-(2-hydroxypropyl)methacrylamide copolymer conjugated with pirarubicin via an acid-cleavable linkage. J. Control. Release.

[14] Hutchinson L. (2010). Breast cancer: Challenges, controversies, breakthroughs. Nat. Rev. Clin. Oncol..

[15] Forman D., Ferlay J., Stewart B.W., Wild C.P. (2014). The global and regional burden of cancer. World Cancer Report.

[16] Murray P.J., Allen J.E., Biswas S.K., Fisher E.A., Gilroy D.W., Goerdt S., Gordon S., Hamilton J.A., Ivashkiv L.B., Lawrence T. (2014). Macrophage activation and polarization: Nomenclature and experimental guidelines. Immunity.

[17] Chen W., Tan H., Liu Q., Zheng X., Liu Y., Xu L. (2019). A Review: The Bioactivities and Pharmacological Applications of *Phellinus linteus*. Molecules.

[18] Kim G.Y., Lee J.Y., Lee J.O., Rru C.H., Choi B.T., Jeong Y.K., Lee K.W., Jeong S.C., Choi Y.H. (2006). Partial characterization and imunostimulatory effect of a novel polysaccharide–protein complex extracted from *Phellinus linteus*. Biosci. Biotechnol. Biochem..

[19] Suabjakyong P., Nishimura K., Toida T., Van Griensven L.J. (2015). Structural characterization and immunomodulatory effects of polysaccharides from *Phellinus linteus* and *Phellinus igniarius* on the IL-6/IL-10 cytokine balance of the mouse macrophage cell lines (RAW264.7). Food Funct..

[20] Kim G.Y., Park S.K., Lee M.K., Lee S.H., Oh Y.H., Kwak J.Y., Yoon S., Lee J.D., Park Y.M. (2003). Proteoglycan isolated from *Phellinus linteu*s activates murine B lymphocytes via protein kinase C and protein tyrosine kinase. Int. Immunopharmacol..

[21] Oh G.S., Lee M.S., Pae H.K., Kwon J., Lee S.S., Jeong J.G., Shin M.K., Kwon T.O., Chung H.T. (2006). Effects of oral administration of *Phellinus linteu*s on the production of Th1- and Th2-type cytokines in mice. Immunopharmacol. Immunotoxicol..

[22] Lin C.J., Lien H.M., Lin H.J., Huang C.L., Kao M.C., Chen Y.A., Wang C.K., Chang H.Y., Chang Y.K., Wu H.S. (2016). Modulation of T cell response by *Phellinus linteus*. J. Biosci. Bioeng..

[23] Calabrese E.J., Baldwin L.A. (2001). U-shaped dose-responses in biology, toxicology, and public health. Annu. Rev. Public Health.

